# 9-[(2-Hy­droxy­benzyl­idene)amino]-11-(2-hy­droxy­phen­yl)-10,13-diphenyl-8-oxa-12-azoniatricyclo­[7.3.1.0^2,7^]trideca-2(7),3,5-triene acetate ethanol disolvate

**DOI:** 10.1107/S160053681100376X

**Published:** 2011-02-05

**Authors:** Le Tuan Anh, Truong Hong Hieu, Anatoly T. Soldatenkov, Svetlana A. Soldatova, Victor N. Khrustalev

**Affiliations:** a Department of Chemistry, Vietnam National University, 144 Xuan Thuy, Cau Giay, Hanoi, Vietnam; b Organic Chemistry Department, Russian Peoples Friendship University, Miklukho-Maklaya St 6, Moscow 117198, Russian Federation; c X-Ray Structural Centre, A. N. Nesmeyanov Institute of Organoelement Compounds, Russian Academy of Sciences, 28 Vavilov St, B-334, Moscow 119991, Russian Federation

## Abstract

The title compound, C_36_H_31_N_2_O_3_^+^,C_2_H_3_O_2_^−^·2C_2_H_5_OH, the product of a domino condensation of dibenzyl ketone with salicylic aldehyde and ammonium acetate, crystallized as the ethanol disolvate. The cation of the salt comprises a fused tricyclic system containing three six-membered rings (piperidine, dihydro-2*H*-pyran and benzene). The piperidine ring has the usual chair conformation, while the dihydro­pyran ring adopts a slightly distorted sofa conformation. In the crystal, there are six (one intra- and five inter­molecular) independent hydrogen-bonding inter­actions: the inter­molecular hydrogen bonds link the cations and anions and ethanol solvent mol­ecules into ribbons along [001]. The ribbons are stacked along the *a* axis.

## Related literature

For general background to the method proposed by our group for obtaining 2-oxa-6-aza­benzobicyclo­nona­nes using commercially available dibenzyl ketone, salicylic aldehyde and ammonium acetate as starting materials, see: Baliah *et al.* (1983 ▶); Soldatenkov *et al.* (1996 ▶); Le Tuan Anh *et al.* (2008 ▶). For related compounds, see: Soldatenkov *et al.* (2002 ▶, 2010 ▶).
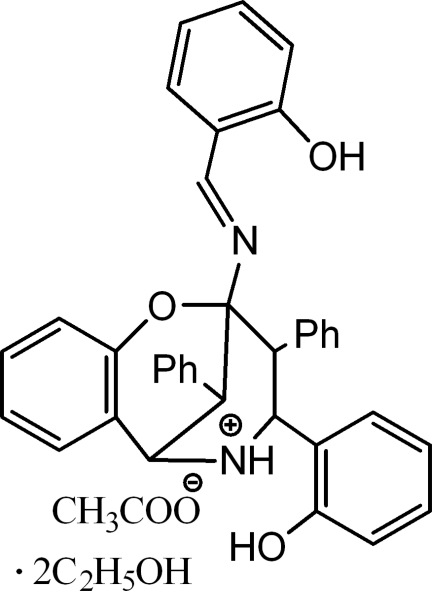


## Experimental

### 

#### Crystal data

C_36_H_31_N_2_O_3_^+^·C_2_H_3_O_2_^−^·2C_2_H_6_O
*M* *_r_* = 690.81
Monoclinic, 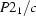

*a* = 13.5464 (10) Å
*b* = 20.1124 (15) Å
*c* = 14.2535 (11) Å
β = 105.118 (2)°
*V* = 3749.0 (5) Å^3^
*Z* = 4
Mo *K*α radiation
μ = 0.08 mm^−1^
*T* = 100 K
0.28 × 0.15 × 0.13 mm

#### Data collection

Bruker APEXII CCD diffractometer
Absorption correction: multi-scan (*SADABS*; Sheldrick, 2003 ▶) *T*_min_ = 0.977, *T*_max_ = 0.989
35569 measured reflections
7399 independent reflections
4951 reflections with *I* > 2σ(*I*)
*R*_int_ = 0.062

#### Refinement

*R*[*F* ^2^ > 2σ(*F* ^2^)] = 0.044
*wR*(*F* ^2^) = 0.107
*S* = 1.01
7399 reflections
463 parameters
H-atom parameters constrained
Δρ_max_ = 0.23 e Å^−3^
Δρ_min_ = −0.24 e Å^−3^

### 

Data collection: *APEX2* (Bruker, 2005 ▶); cell refinement: *SAINT-Plus* (Bruker, 2001 ▶); data reduction: *SAINT-Plus*; program(s) used to solve structure: *SHELXTL* (Sheldrick, 2008 ▶); program(s) used to refine structure: *SHELXTL*; molecular graphics: *SHELXTL*; software used to prepare material for publication: *SHELXTL*.

## Supplementary Material

Crystal structure: contains datablocks global, I. DOI: 10.1107/S160053681100376X/rk2264sup1.cif

Structure factors: contains datablocks I. DOI: 10.1107/S160053681100376X/rk2264Isup2.hkl

Additional supplementary materials:  crystallographic information; 3D view; checkCIF report

## Figures and Tables

**Table 1:** Hydrogen-bond geometry (Å, °)

*D*—H⋯*A*	*D*—H	H⋯*A*	*D*⋯*A*	*D*—H⋯*A*
O1—H1*O*⋯N1	0.94	1.73	2.608 (2)	154
O2—H2*O*⋯O3^i^	0.97	1.67	2.637 (2)	177
O5—H5*O*⋯O6^ii^	0.97	1.69	2.651 (2)	174
O6—H6*O*⋯O4	0.98	1.65	2.617 (2)	173
N12—H12*A*⋯O3	0.93	1.77	2.697 (2)	172
N12—H12*B*⋯O5	0.94	1.77	2.709 (2)	173

Symmetry codes: (i) 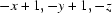; (ii) 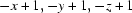.

**Fig. 1.**
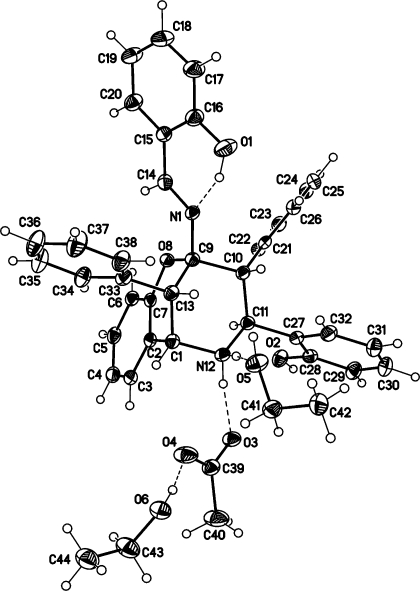
Molecular structure of I with the atom numbering scheme. Displacement ellipsoids are shown at the 50% probability level. H atoms are presented as a small spheres of arbitrary radius. Dashed lines indicate hydrogen bonds.

**Fig. 2.**
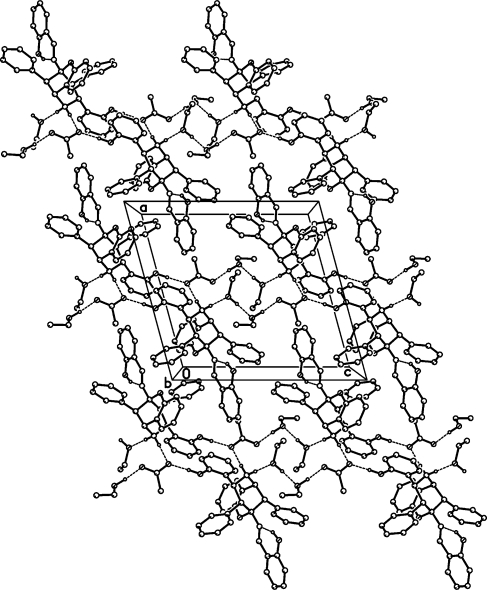
Crystal packing of I. Dashed lines indicate hydrogen bonds.

**Fig. 3.**
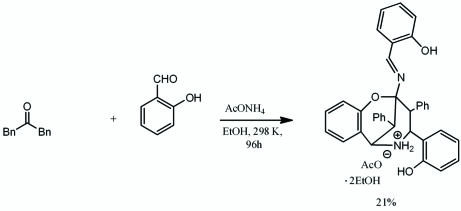
Domino condensation of dibenzyl ketone with salicylic aldehyde and ammonium acetate.

## supplementary crystallographic information

### Comment

Recently our group has found an efficient method of the one-step synthesis of potentially bioactive substances having oxazocine skeletal structure. These molecules are formed by domino condensation from commercially available dibenzyl ketone, salicylic aldehyde and ammonium acetate as starting materials (Soldatenkov *et al.*, 2010). The key step of this condensation is Petrenko–Kritchenko reaction (Baliah *et al.*, 1983) leading to the formation of the substituted γ-piperidone (Le Tuan Anh *et al.*, 2008), which then reacts with the excess of ammonium acetate and aldehyde. This work reports the structural characterization of a product of such reaction - 2-oxa-6-aza-3,4-benzobicyclo[3.3.1^1,5^]nonan-6-ium acetate (I).

Compound I crystallizes as diethanol solvate, *i.e.*, C_38_H_34_N_2_O_5_^.^2(C_2_H_6_O). The cation of the salt I comprises a fused tricyclic system containing three six-membered rings (piperidine, dihydro-2*H*-pyran and benzene) (Fig. 1). The piperidine ring has the usual *chair* conformation, while the dihydropyran ring adopts the slightly distorted *sofa* conformation (the C13 carbon atom deviates from the plane passed through the other atoms of the ring by 0.691 (2) Å). The phenyl substituents at the C10 and C11 carbon atoms occupy the sterically favorable equatorial positions, whereas the phenyl substituent at the C13 carbon atom is axially disposed.

The cation of I possesses four asymmetric centers at the C1, C10, C11, and C13 carbon atoms and can have potentially numerous diastereomers. The crystal of I is racemic and consists of enantiomeric pairs with the following relative configuration of the centers: *rac*-1*S**,10*R**,11*S**, 13*S**.

In the crystal, there are six (one intra- and five intermolecular) independent hydrogen bonding interactions (Table 1). The intermolecular hydrogen bonds link the cations and anions of I and ethanol solvate molecules into ribbons along the direction [0 0 1] (Fig. 2). The crystal packing of the ribbons is stacked along the *a* axis.

### Experimental

Ammonium acetate (4.0 g, 52 mmol) was added to a solution of dibenzyl ketone (2.1 g, 10 mmol) and salicylic aldehyde (3.66 g, 30 mmol) in ethanol (50 ml) (Fig. 3). The reaction mixture was stirred for 96 h at 293 K (monitoring by *TLC* until disappearance of the starting ketone spot). At the end of the reaction, the formed precipitate was filtered off, one half of the mother liquid solvent removed under reduced pressure and the residue was cooled to give 1.45 g of light-yellow crystals of I. Yield is 21%. *M*.p. = 451–453 K. IR (KBr), ν/cm^-1^: 1623, 1748, 3405, 3460. ^1^H NMR (DMSO-*d*_6_, 400 MHz, 300 K): δ = 1.08 (t, 6H, CH_3_CH_2_O, J = 6.8), 3.30 (s, 3H, CH_3_CO), 3.47 (q, 4H, CH_3_CH_2_O, J = 6.8), 3.77 (d, 1H, H8, J_7.8_ = 9.0), 4.23 (d, 1H, H9, J_5,9_ = 1.5), 4.32 (d, 1H, H7, J_7,8_= 9.0), 4.41 (br, 4H, 2(*Alk*)OH, ^+^NH_2_), 4.48 (d, 1H, H5, J_5,9_ = 1.5), 6.41–7.50 (br m, 22H, H_arom_), 7.94 (s, 1H, N=CH), 10.63 (br, 1H, (*Ar*)OH), 12.48 (s, 1H, (*Ar*)OH). Anal. Calcd. for C_42_H_46_N_2_O_7_: C, 73.04; H, 6.67; N, 4.06. Found: C, 73.13; H, 6.79; N, 4.23.

### Refinement

The hydrogen atoms of the hydroxy and amino groups were localized in the difference Fourier map and included in the refinement with fixed positional and isotropic displacement parameters [*U*_iso_(H) = 1.5*U*_eq_(O) and 1.2*U*_eq_(N)]. The other hydrogen atoms were placed in calculated positions with C—H = 0.95–1.00Å and refined in the riding model with fixed isotropic displacement parameters [*U*_iso_(H) = 1.5*U*_eq_(C) for CH_3_-groups and *U*_iso_(H) = 1.2*U*_eq_(C) for the other groups].

### Crystal data

**Table:**

C_36_H_31_N_2_O_3_^+^·C_2_H_3_O_2_^−^·2C_2_H_6_O	*F*(000) = 1472
*M**_r_* = 690.81	*D*_x_ = 1.224 Mg m^−^^3^
Monoclinic, *P*2_1_/*c*	Melting point = 451–453 K
Hall symbol: -P 2ybc	Mo *K*α radiation, λ = 0.71073 Å
*a* = 13.5464 (10) Å	Cell parameters from 4349 reflections
*b* = 20.1124 (15) Å	θ = 2.5–23.7°
*c* = 14.2535 (11) Å	µ = 0.08 mm^−^^1^
β = 105.118 (2)°	*T* = 100 K
*V* = 3749.0 (5) Å^3^	Prism, light-yellow
*Z* = 4	0.28 × 0.15 × 0.13 mm

### Data collection

**Table:**

Bruker APEXII CCD diffractometer	7399 independent reflections
Radiation source: fine-focus sealed tube	4951 reflections with *I* > 2σ(*I*)
graphite	*R*_int_ = 0.062
φ and ω scans	θ_max_ = 26.1°, θ_min_ = 1.6°
Absorption correction: multi-scan (*SADABS*; Sheldrick, 2003)	*h* = −16→16
*T*_min_ = 0.977, *T*_max_ = 0.989	*k* = −24→24
35569 measured reflections	*l* = −17→17

### Refinement

**Table:**

Refinement on *F*^2^	Primary atom site location: structure-invariant direct methods
Least-squares matrix: full	Secondary atom site location: difference Fourier map
*R*[*F*^2^ > 2σ(*F*^2^)] = 0.044	Hydrogen site location: difference Fourier map
*wR*(*F*^2^) = 0.107	H-atom parameters constrained
*S* = 1.01	*w* = 1/[σ^2^(*F*_o_^2^) + (0.044*P*)^2^ + 0.8*P*] where *P* = (*F*_o_^2^ + 2*F*_c_^2^)/3
7399 reflections	(Δ/σ)_max_ = 0.001
463 parameters	Δρ_max_ = 0.23 e Å^−^^3^
0 restraints	Δρ_min_ = −0.24 e Å^−^^3^

### Special details

**Table:**

Geometry. All s.u.'s (except the s.u. in the dihedral angle between two l.s. planes) are estimated using the full covariance matrix. The cell s.u.'s are taken into account individually in the estimation of s.u.'s in distances, angles and torsion angles; correlations between s.u.'s in cell parameters are only used when they are defined by crystal symmetry. An approximate (isotropic) treatment of cell s.u.'s is used for estimating s.u.'s involving l.s. planes.
Refinement. Refinement of *F*^2^ against ALL reflections. The weighted *R*-factor w*R* and goodness of fit *S* are based on *F*^2^, conventional *R*-factors *R* are based on *F*, with *F* set to zero for negative *F*^2^. The threshold expression of *F*^2^ > 2σ(*F*^2^) is used only for calculating *R*-factors(gt) *etc*. and is not relevant to the choice of reflections for refinement. *R*-factors based on *F*^2^ are statistically about twice as large as those based on *F*, and *R*-factors based on ALL data will be even larger.

### Fractional atomic coordinates and isotropic or equivalent isotropic displacement parameters (Å^2^)

**Table:**

	*x*	*y*	*z*	*U*_iso_*/*U*_eq_	
C1	0.31447 (13)	0.45725 (9)	0.26034 (13)	0.0241 (4)	
H1	0.3562	0.4309	0.3162	0.029*	
C2	0.27020 (13)	0.41161 (8)	0.17702 (13)	0.0238 (4)	
C3	0.31983 (14)	0.35414 (9)	0.15984 (14)	0.0286 (4)	
H3	0.3835	0.3423	0.2031	0.034*	
C4	0.27796 (14)	0.31398 (9)	0.08085 (15)	0.0332 (5)	
H4	0.3128	0.2749	0.0698	0.040*	
C5	0.18468 (14)	0.33100 (9)	0.01773 (14)	0.0308 (4)	
H5	0.1562	0.3036	−0.0370	0.037*	
C6	0.13298 (13)	0.38735 (8)	0.03375 (13)	0.0253 (4)	
H6	0.0687	0.3985	−0.0089	0.030*	
C7	0.17631 (13)	0.42725 (8)	0.11278 (12)	0.0224 (4)	
O8	0.11919 (8)	0.48161 (5)	0.12638 (8)	0.0223 (3)	
C9	0.16377 (13)	0.52919 (8)	0.20072 (12)	0.0218 (4)	
C10	0.22947 (12)	0.58026 (8)	0.16205 (13)	0.0229 (4)	
H10	0.2529	0.6143	0.2143	0.027*	
C11	0.32643 (12)	0.54871 (8)	0.14383 (12)	0.0222 (4)	
H11	0.3046	0.5182	0.0867	0.027*	
N12	0.38139 (10)	0.50797 (7)	0.22941 (10)	0.0226 (3)	
H12A	0.4342	0.4854	0.2119	0.027*	
H12B	0.4088	0.5347	0.2840	0.027*	
C13	0.23087 (12)	0.49527 (8)	0.29266 (12)	0.0235 (4)	
H13	0.2660	0.5317	0.3365	0.028*	
N1	0.08334 (10)	0.56563 (7)	0.22658 (10)	0.0232 (3)	
C14	−0.00930 (13)	0.54651 (9)	0.20077 (13)	0.0256 (4)	
H14	−0.0258	0.5075	0.1623	0.031*	
C15	−0.09103 (13)	0.58249 (9)	0.22817 (13)	0.0260 (4)	
C16	−0.07164 (14)	0.64102 (10)	0.28307 (14)	0.0335 (5)	
O1	0.02500 (10)	0.66495 (7)	0.31689 (12)	0.0508 (4)	
H1O	0.0648	0.6343	0.2931	0.076*	
C17	−0.15130 (15)	0.67540 (10)	0.30517 (16)	0.0420 (5)	
H17	−0.1381	0.7155	0.3416	0.050*	
C18	−0.24953 (15)	0.65141 (11)	0.27434 (15)	0.0411 (5)	
H18	−0.3037	0.6752	0.2899	0.049*	
C19	−0.27084 (15)	0.59328 (10)	0.22123 (15)	0.0392 (5)	
H19	−0.3388	0.5768	0.2012	0.047*	
C20	−0.19200 (14)	0.55965 (10)	0.19783 (14)	0.0333 (5)	
H20	−0.2064	0.5201	0.1603	0.040*	
C21	0.16999 (12)	0.61709 (8)	0.07242 (13)	0.0239 (4)	
C22	0.15273 (13)	0.58968 (9)	−0.01999 (13)	0.0264 (4)	
H22	0.1762	0.5460	−0.0273	0.032*	
C23	0.10186 (13)	0.62515 (9)	−0.10155 (14)	0.0301 (4)	
H23	0.0917	0.6059	−0.1642	0.036*	
C24	0.06575 (14)	0.68840 (10)	−0.09224 (15)	0.0347 (5)	
H24	0.0309	0.7127	−0.1482	0.042*	
C25	0.08082 (14)	0.71584 (9)	−0.00093 (16)	0.0366 (5)	
H25	0.0554	0.7591	0.0060	0.044*	
C26	0.13278 (13)	0.68080 (9)	0.08103 (15)	0.0298 (4)	
H26	0.1431	0.7004	0.1435	0.036*	
C27	0.39849 (12)	0.59989 (9)	0.12015 (13)	0.0241 (4)	
C28	0.43138 (13)	0.59222 (9)	0.03550 (13)	0.0257 (4)	
O2	0.39434 (9)	0.54015 (6)	−0.02342 (9)	0.0311 (3)	
H2O	0.4183	0.5416	−0.0816	0.047*	
C29	0.49980 (14)	0.63804 (10)	0.01430 (14)	0.0340 (5)	
H29	0.5235	0.6326	−0.0423	0.041*	
C30	0.53299 (15)	0.69114 (10)	0.07520 (16)	0.0394 (5)	
H30	0.5798	0.7220	0.0603	0.047*	
C31	0.49895 (15)	0.70016 (10)	0.15790 (15)	0.0361 (5)	
H31	0.5206	0.7376	0.1988	0.043*	
C32	0.43302 (13)	0.65386 (9)	0.18001 (14)	0.0293 (4)	
H32	0.4109	0.6592	0.2376	0.035*	
C33	0.17145 (13)	0.45525 (9)	0.35001 (13)	0.0268 (4)	
C34	0.12157 (14)	0.39565 (10)	0.31830 (14)	0.0332 (5)	
H34	0.1256	0.3769	0.2582	0.040*	
C35	0.06608 (16)	0.36341 (11)	0.37368 (15)	0.0427 (5)	
H35	0.0325	0.3227	0.3513	0.051*	
C36	0.05916 (17)	0.39003 (12)	0.46139 (16)	0.0477 (6)	
H36	0.0200	0.3682	0.4987	0.057*	
C37	0.10967 (17)	0.44863 (11)	0.49416 (16)	0.0464 (6)	
H37	0.1066	0.4667	0.5549	0.056*	
C38	0.16484 (15)	0.48112 (10)	0.43867 (14)	0.0357 (5)	
H38	0.1986	0.5217	0.4615	0.043*	
O5	0.46371 (10)	0.57558 (7)	0.39577 (9)	0.0374 (3)	
H5O	0.4207	0.5946	0.4335	0.056*	
C41	0.56962 (14)	0.57329 (10)	0.44520 (15)	0.0351 (5)	
H41A	0.6090	0.5584	0.3995	0.042*	
H41B	0.5804	0.5405	0.4987	0.042*	
C42	0.60860 (16)	0.63970 (10)	0.48591 (17)	0.0454 (6)	
H42A	0.6826	0.6371	0.5146	0.068*	
H42B	0.5747	0.6526	0.5360	0.068*	
H42C	0.5941	0.6729	0.4338	0.068*	
O6	0.66244 (10)	0.37980 (6)	0.50427 (10)	0.0369 (3)	
H6O	0.6265	0.3937	0.4387	0.055*	
C43	0.70231 (18)	0.31419 (11)	0.50708 (16)	0.0468 (6)	
H43A	0.7738	0.3162	0.5021	0.056*	
H43B	0.6617	0.2886	0.4508	0.056*	
C44	0.69941 (17)	0.27955 (11)	0.59883 (16)	0.0474 (6)	
H44A	0.7292	0.2351	0.5997	0.071*	
H44B	0.6284	0.2757	0.6024	0.071*	
H44C	0.7388	0.3051	0.6546	0.071*	
C39	0.59166 (14)	0.42003 (10)	0.25775 (14)	0.0321 (4)	
C40	0.69533 (16)	0.39181 (13)	0.25958 (17)	0.0524 (6)	
H40A	0.7006	0.3464	0.2854	0.079*	
H40B	0.7487	0.4195	0.3010	0.079*	
H40C	0.7040	0.3911	0.1934	0.079*	
O3	0.54470 (9)	0.45292 (6)	0.18438 (9)	0.0316 (3)	
O4	0.55567 (10)	0.41052 (8)	0.32869 (10)	0.0459 (4)	

### Atomic displacement parameters (Å^2^)

**Table:**

	*U*^11^	*U*^22^	*U*^33^	*U*^12^	*U*^13^	*U*^23^
C1	0.0223 (9)	0.0253 (9)	0.0253 (10)	−0.0004 (7)	0.0072 (7)	0.0040 (8)
C2	0.0242 (9)	0.0216 (9)	0.0288 (10)	−0.0005 (7)	0.0125 (8)	0.0047 (7)
C3	0.0266 (10)	0.0247 (10)	0.0386 (11)	0.0017 (7)	0.0159 (8)	0.0058 (8)
C4	0.0349 (11)	0.0221 (10)	0.0505 (13)	−0.0001 (8)	0.0252 (10)	−0.0016 (9)
C5	0.0350 (11)	0.0241 (10)	0.0391 (11)	−0.0062 (8)	0.0200 (9)	−0.0069 (8)
C6	0.0261 (10)	0.0251 (10)	0.0277 (10)	−0.0021 (7)	0.0125 (8)	−0.0005 (8)
C7	0.0257 (9)	0.0186 (9)	0.0282 (10)	−0.0005 (7)	0.0161 (8)	0.0007 (7)
O8	0.0229 (6)	0.0206 (6)	0.0247 (7)	0.0012 (5)	0.0084 (5)	−0.0030 (5)
C9	0.0227 (9)	0.0214 (9)	0.0232 (9)	0.0003 (7)	0.0092 (7)	−0.0042 (7)
C10	0.0204 (9)	0.0220 (9)	0.0281 (10)	−0.0014 (7)	0.0095 (7)	−0.0032 (7)
C11	0.0217 (9)	0.0233 (9)	0.0223 (9)	0.0008 (7)	0.0070 (7)	0.0004 (7)
N12	0.0196 (7)	0.0264 (8)	0.0233 (8)	0.0020 (6)	0.0082 (6)	0.0002 (6)
C13	0.0232 (9)	0.0252 (9)	0.0231 (9)	−0.0017 (7)	0.0077 (7)	−0.0021 (7)
N1	0.0222 (8)	0.0225 (8)	0.0281 (8)	−0.0005 (6)	0.0122 (6)	−0.0022 (6)
C14	0.0298 (10)	0.0239 (9)	0.0257 (10)	−0.0014 (8)	0.0117 (8)	−0.0019 (8)
C15	0.0266 (10)	0.0263 (10)	0.0271 (10)	0.0006 (7)	0.0103 (8)	0.0003 (8)
C16	0.0303 (11)	0.0330 (11)	0.0398 (12)	−0.0020 (8)	0.0138 (9)	−0.0090 (9)
O1	0.0290 (8)	0.0438 (9)	0.0836 (12)	−0.0086 (6)	0.0216 (8)	−0.0343 (8)
C17	0.0369 (12)	0.0369 (12)	0.0552 (14)	0.0023 (9)	0.0178 (10)	−0.0153 (10)
C18	0.0308 (11)	0.0492 (13)	0.0459 (13)	0.0067 (9)	0.0145 (10)	−0.0095 (11)
C19	0.0261 (11)	0.0481 (13)	0.0455 (13)	−0.0007 (9)	0.0130 (9)	−0.0105 (10)
C20	0.0295 (11)	0.0368 (11)	0.0352 (11)	−0.0037 (8)	0.0115 (9)	−0.0082 (9)
C21	0.0168 (9)	0.0237 (9)	0.0329 (10)	−0.0022 (7)	0.0095 (7)	0.0018 (8)
C22	0.0222 (9)	0.0248 (10)	0.0334 (11)	0.0013 (7)	0.0093 (8)	0.0024 (8)
C23	0.0260 (10)	0.0335 (11)	0.0321 (11)	−0.0022 (8)	0.0098 (8)	0.0061 (9)
C24	0.0261 (10)	0.0317 (11)	0.0447 (13)	−0.0012 (8)	0.0066 (9)	0.0136 (9)
C25	0.0313 (11)	0.0211 (10)	0.0584 (15)	0.0014 (8)	0.0134 (10)	0.0061 (9)
C26	0.0266 (10)	0.0234 (10)	0.0414 (12)	−0.0015 (8)	0.0125 (9)	−0.0015 (8)
C27	0.0191 (9)	0.0246 (9)	0.0293 (10)	0.0007 (7)	0.0073 (7)	0.0024 (8)
C28	0.0211 (9)	0.0276 (10)	0.0281 (10)	0.0017 (7)	0.0062 (8)	0.0022 (8)
O2	0.0320 (7)	0.0357 (8)	0.0297 (7)	−0.0059 (6)	0.0151 (6)	−0.0045 (6)
C29	0.0336 (11)	0.0381 (12)	0.0342 (11)	−0.0049 (9)	0.0156 (9)	0.0045 (9)
C30	0.0366 (12)	0.0350 (12)	0.0502 (13)	−0.0092 (9)	0.0179 (10)	0.0060 (10)
C31	0.0329 (11)	0.0288 (10)	0.0461 (13)	−0.0074 (8)	0.0094 (9)	−0.0046 (9)
C32	0.0253 (10)	0.0318 (11)	0.0322 (11)	−0.0015 (8)	0.0099 (8)	−0.0021 (8)
C33	0.0237 (9)	0.0326 (10)	0.0248 (10)	−0.0006 (8)	0.0075 (8)	0.0018 (8)
C34	0.0349 (11)	0.0405 (12)	0.0261 (10)	−0.0098 (9)	0.0113 (8)	−0.0003 (9)
C35	0.0465 (13)	0.0471 (13)	0.0373 (12)	−0.0188 (10)	0.0163 (10)	−0.0013 (10)
C36	0.0536 (14)	0.0591 (15)	0.0381 (13)	−0.0189 (11)	0.0259 (11)	0.0012 (11)
C37	0.0578 (14)	0.0557 (15)	0.0352 (12)	−0.0153 (11)	0.0289 (11)	−0.0087 (11)
C38	0.0383 (11)	0.0382 (12)	0.0351 (11)	−0.0071 (9)	0.0178 (9)	−0.0051 (9)
O5	0.0285 (7)	0.0497 (9)	0.0331 (8)	−0.0006 (6)	0.0064 (6)	−0.0104 (7)
C41	0.0284 (10)	0.0357 (11)	0.0389 (12)	−0.0008 (8)	0.0045 (9)	−0.0025 (9)
C42	0.0394 (12)	0.0343 (12)	0.0590 (15)	−0.0062 (9)	0.0067 (11)	0.0009 (11)
O6	0.0375 (8)	0.0375 (8)	0.0361 (8)	0.0077 (6)	0.0102 (6)	−0.0005 (6)
C43	0.0541 (14)	0.0419 (13)	0.0465 (14)	0.0159 (11)	0.0171 (11)	0.0013 (11)
C44	0.0503 (14)	0.0439 (13)	0.0507 (14)	0.0129 (10)	0.0177 (11)	0.0050 (11)
C39	0.0282 (10)	0.0365 (11)	0.0344 (11)	0.0031 (8)	0.0133 (9)	0.0011 (9)
C40	0.0419 (13)	0.0696 (17)	0.0522 (15)	0.0227 (12)	0.0238 (11)	0.0142 (12)
O3	0.0268 (7)	0.0401 (8)	0.0302 (7)	0.0057 (6)	0.0117 (6)	0.0035 (6)
O4	0.0382 (8)	0.0664 (11)	0.0376 (9)	0.0189 (7)	0.0181 (7)	0.0160 (7)

### Geometric parameters (Å, °)

**Table:**

C1—C2	1.497 (2)	C24—C25	1.379 (3)
C1—N12	1.505 (2)	C24—H24	0.9500
C1—C13	1.533 (2)	C25—C26	1.388 (3)
C1—H1	1.0000	C25—H25	0.9500
C2—C3	1.391 (2)	C26—H26	0.9500
C2—C7	1.396 (2)	C27—C32	1.384 (2)
C3—C4	1.382 (3)	C27—C28	1.400 (2)
C3—H3	0.9500	C28—O2	1.354 (2)
C4—C5	1.389 (3)	C28—C29	1.395 (2)
C4—H4	0.9500	O2—H2O	0.9659
C5—C6	1.382 (2)	C29—C30	1.376 (3)
C5—H5	0.9500	C29—H29	0.9500
C6—C7	1.383 (2)	C30—C31	1.385 (3)
C6—H6	0.9500	C30—H30	0.9500
C7—O8	1.3820 (19)	C31—C32	1.382 (3)
O8—C9	1.438 (2)	C31—H31	0.9500
C9—N1	1.439 (2)	C32—H32	0.9500
C9—C13	1.545 (2)	C33—C38	1.391 (3)
C9—C10	1.551 (2)	C33—C34	1.392 (3)
C10—C21	1.514 (2)	C34—C35	1.385 (3)
C10—C11	1.541 (2)	C34—H34	0.9500
C10—H10	1.0000	C35—C36	1.385 (3)
C11—N12	1.497 (2)	C35—H35	0.9500
C11—C27	1.516 (2)	C36—C37	1.382 (3)
C11—H11	1.0000	C36—H36	0.9500
N12—H12A	0.9347	C37—C38	1.386 (3)
N12—H12B	0.9381	C37—H37	0.9500
C13—C33	1.519 (2)	C38—H38	0.9500
C13—H13	1.0000	O5—C41	1.425 (2)
N1—C14	1.272 (2)	O5—H5O	0.9692
C14—C15	1.459 (2)	C41—C42	1.497 (3)
C14—H14	0.9500	C41—H41A	0.9900
C15—C16	1.400 (3)	C41—H41B	0.9900
C15—C20	1.400 (3)	C42—H42A	0.9800
C16—O1	1.360 (2)	C42—H42B	0.9800
C16—C17	1.385 (3)	C42—H42C	0.9800
O1—H1O	0.9389	O6—C43	1.422 (2)
C17—C18	1.375 (3)	O6—H6O	0.9752
C17—H17	0.9500	C43—C44	1.491 (3)
C18—C19	1.382 (3)	C43—H43A	0.9900
C18—H18	0.9500	C43—H43B	0.9900
C19—C20	1.377 (3)	C44—H44A	0.9800
C19—H19	0.9500	C44—H44B	0.9800
C20—H20	0.9500	C44—H44C	0.9800
C21—C22	1.390 (3)	C39—O4	1.246 (2)
C21—C26	1.394 (2)	C39—O3	1.261 (2)
C22—C23	1.385 (2)	C39—C40	1.509 (3)
C22—H22	0.9500	C40—H40A	0.9800
C23—C24	1.382 (3)	C40—H40B	0.9800
C23—H23	0.9500	C40—H40C	0.9800
			
C2—C1—N12	109.30 (14)	C24—C23—C22	120.32 (19)
C2—C1—C13	111.69 (14)	C24—C23—H23	119.8
N12—C1—C13	107.37 (13)	C22—C23—H23	119.8
C2—C1—H1	109.5	C25—C24—C23	119.36 (18)
N12—C1—H1	109.5	C25—C24—H24	120.3
C13—C1—H1	109.5	C23—C24—H24	120.3
C3—C2—C7	118.13 (16)	C24—C25—C26	120.58 (18)
C3—C2—C1	122.50 (16)	C24—C25—H25	119.7
C7—C2—C1	119.36 (15)	C26—C25—H25	119.7
C4—C3—C2	120.99 (18)	C25—C26—C21	120.53 (18)
C4—C3—H3	119.5	C25—C26—H26	119.7
C2—C3—H3	119.5	C21—C26—H26	119.7
C3—C4—C5	119.63 (17)	C32—C27—C28	119.04 (16)
C3—C4—H4	120.2	C32—C27—C11	121.92 (16)
C5—C4—H4	120.2	C28—C27—C11	119.03 (15)
C6—C5—C4	120.63 (18)	O2—C28—C29	122.43 (16)
C6—C5—H5	119.7	O2—C28—C27	118.04 (15)
C4—C5—H5	119.7	C29—C28—C27	119.53 (17)
C5—C6—C7	118.99 (17)	C28—O2—H2O	110.8
C5—C6—H6	120.5	C30—C29—C28	120.17 (18)
C7—C6—H6	120.5	C30—C29—H29	119.9
O8—C7—C6	116.05 (15)	C28—C29—H29	119.9
O8—C7—C2	122.28 (15)	C29—C30—C31	120.79 (18)
C6—C7—C2	121.61 (16)	C29—C30—H30	119.6
C7—O8—C9	119.13 (13)	C31—C30—H30	119.6
O8—C9—N1	109.09 (13)	C32—C31—C30	118.95 (18)
O8—C9—C13	111.85 (13)	C32—C31—H31	120.5
N1—C9—C13	108.96 (13)	C30—C31—H31	120.5
O8—C9—C10	110.38 (13)	C31—C32—C27	121.48 (18)
N1—C9—C10	107.26 (13)	C31—C32—H32	119.3
C13—C9—C10	109.18 (13)	C27—C32—H32	119.3
C21—C10—C11	110.40 (14)	C38—C33—C34	118.44 (17)
C21—C10—C9	113.29 (14)	C38—C33—C13	117.35 (16)
C11—C10—C9	112.37 (14)	C34—C33—C13	124.20 (16)
C21—C10—H10	106.8	C35—C34—C33	120.51 (18)
C11—C10—H10	106.8	C35—C34—H34	119.7
C9—C10—H10	106.8	C33—C34—H34	119.7
N12—C11—C27	109.93 (13)	C34—C35—C36	120.53 (19)
N12—C11—C10	110.66 (13)	C34—C35—H35	119.7
C27—C11—C10	112.63 (14)	C36—C35—H35	119.7
N12—C11—H11	107.8	C37—C36—C35	119.38 (19)
C27—C11—H11	107.8	C37—C36—H36	120.3
C10—C11—H11	107.8	C35—C36—H36	120.3
C11—N12—C1	113.59 (13)	C36—C37—C38	120.18 (19)
C11—N12—H12A	107.5	C36—C37—H37	119.9
C1—N12—H12A	108.1	C38—C37—H37	119.9
C11—N12—H12B	111.5	C37—C38—C33	120.95 (19)
C1—N12—H12B	106.5	C37—C38—H38	119.5
H12A—N12—H12B	109.5	C33—C38—H38	119.5
C33—C13—C1	115.72 (14)	C41—O5—H5O	114.3
C33—C13—C9	114.42 (14)	O5—C41—C42	111.77 (16)
C1—C13—C9	106.41 (13)	O5—C41—H41A	109.3
C33—C13—H13	106.6	C42—C41—H41A	109.3
C1—C13—H13	106.6	O5—C41—H41B	109.3
C9—C13—H13	106.6	C42—C41—H41B	109.3
C14—N1—C9	121.86 (15)	H41A—C41—H41B	107.9
N1—C14—C15	122.22 (16)	C41—C42—H42A	109.5
N1—C14—H14	118.9	C41—C42—H42B	109.5
C15—C14—H14	118.9	H42A—C42—H42B	109.5
C16—C15—C20	118.30 (17)	C41—C42—H42C	109.5
C16—C15—C14	121.47 (16)	H42A—C42—H42C	109.5
C20—C15—C14	120.21 (16)	H42B—C42—H42C	109.5
O1—C16—C17	118.54 (17)	C43—O6—H6O	112.5
O1—C16—C15	121.25 (16)	O6—C43—C44	111.19 (17)
C17—C16—C15	120.21 (18)	O6—C43—H43A	109.4
C16—O1—H1O	103.3	C44—C43—H43A	109.4
C18—C17—C16	119.89 (19)	O6—C43—H43B	109.4
C18—C17—H17	120.1	C44—C43—H43B	109.4
C16—C17—H17	120.1	H43A—C43—H43B	108.0
C17—C18—C19	121.22 (18)	C43—C44—H44A	109.5
C17—C18—H18	119.4	C43—C44—H44B	109.5
C19—C18—H18	119.4	H44A—C44—H44B	109.5
C20—C19—C18	118.95 (18)	C43—C44—H44C	109.5
C20—C19—H19	120.5	H44A—C44—H44C	109.5
C18—C19—H19	120.5	H44B—C44—H44C	109.5
C19—C20—C15	121.41 (18)	O4—C39—O3	122.28 (17)
C19—C20—H20	119.3	O4—C39—C40	119.18 (18)
C15—C20—H20	119.3	O3—C39—C40	118.54 (17)
C22—C21—C26	118.20 (17)	C39—C40—H40A	109.5
C22—C21—C10	121.76 (15)	C39—C40—H40B	109.5
C26—C21—C10	120.03 (16)	H40A—C40—H40B	109.5
C23—C22—C21	121.00 (17)	C39—C40—H40C	109.5
C23—C22—H22	119.5	H40A—C40—H40C	109.5
C21—C22—H22	119.5	H40B—C40—H40C	109.5
			
N12—C1—C2—C3	88.45 (19)	C20—C15—C16—O1	178.30 (18)
C13—C1—C2—C3	−152.89 (16)	C14—C15—C16—O1	−3.1 (3)
N12—C1—C2—C7	−90.60 (18)	C20—C15—C16—C17	−0.8 (3)
C13—C1—C2—C7	28.1 (2)	C14—C15—C16—C17	177.75 (18)
C7—C2—C3—C4	0.7 (3)	O1—C16—C17—C18	−178.2 (2)
C1—C2—C3—C4	−178.39 (16)	C15—C16—C17—C18	1.0 (3)
C2—C3—C4—C5	−0.3 (3)	C16—C17—C18—C19	−0.1 (3)
C3—C4—C5—C6	−0.6 (3)	C17—C18—C19—C20	−1.0 (3)
C4—C5—C6—C7	1.1 (3)	C18—C19—C20—C15	1.2 (3)
C5—C6—C7—O8	−178.01 (15)	C16—C15—C20—C19	−0.3 (3)
C5—C6—C7—C2	−0.6 (3)	C14—C15—C20—C19	−178.86 (18)
C3—C2—C7—O8	176.98 (15)	C11—C10—C21—C22	44.7 (2)
C1—C2—C7—O8	−3.9 (2)	C9—C10—C21—C22	−82.32 (19)
C3—C2—C7—C6	−0.2 (2)	C11—C10—C21—C26	−133.91 (16)
C1—C2—C7—C6	178.88 (15)	C9—C10—C21—C26	99.07 (18)
C6—C7—O8—C9	−173.46 (14)	C26—C21—C22—C23	1.3 (2)
C2—C7—O8—C9	9.2 (2)	C10—C21—C22—C23	−177.29 (16)
C7—O8—C9—N1	−158.67 (13)	C21—C22—C23—C24	−1.0 (3)
C7—O8—C9—C13	−38.05 (18)	C22—C23—C24—C25	0.0 (3)
C7—O8—C9—C10	83.72 (17)	C23—C24—C25—C26	0.8 (3)
O8—C9—C10—C21	57.51 (18)	C24—C25—C26—C21	−0.4 (3)
N1—C9—C10—C21	−61.23 (18)	C22—C21—C26—C25	−0.6 (3)
C13—C9—C10—C21	−179.16 (14)	C10—C21—C26—C25	178.05 (16)
O8—C9—C10—C11	−68.47 (17)	N12—C11—C27—C32	−70.3 (2)
N1—C9—C10—C11	172.79 (13)	C10—C11—C27—C32	53.6 (2)
C13—C9—C10—C11	54.86 (18)	N12—C11—C27—C28	109.50 (17)
C21—C10—C11—N12	−175.72 (13)	C10—C11—C27—C28	−126.60 (17)
C9—C10—C11—N12	−48.19 (18)	C32—C27—C28—O2	−178.12 (15)
C21—C10—C11—C27	60.78 (18)	C11—C27—C28—O2	2.1 (2)
C9—C10—C11—C27	−171.69 (14)	C32—C27—C28—C29	1.5 (3)
C27—C11—N12—C1	177.98 (13)	C11—C27—C28—C29	−178.31 (16)
C10—C11—N12—C1	52.95 (18)	O2—C28—C29—C30	178.19 (17)
C2—C1—N12—C11	58.31 (18)	C27—C28—C29—C30	−1.4 (3)
C13—C1—N12—C11	−63.00 (17)	C28—C29—C30—C31	−0.3 (3)
C2—C1—C13—C33	74.96 (19)	C29—C30—C31—C32	1.8 (3)
N12—C1—C13—C33	−165.24 (14)	C30—C31—C32—C27	−1.7 (3)
C2—C1—C13—C9	−53.37 (18)	C28—C27—C32—C31	0.1 (3)
N12—C1—C13—C9	66.44 (16)	C11—C27—C32—C31	179.82 (17)
O8—C9—C13—C33	−69.97 (18)	C1—C13—C33—C38	127.54 (18)
N1—C9—C13—C33	50.72 (19)	C9—C13—C33—C38	−108.19 (18)
C10—C9—C13—C33	167.57 (14)	C1—C13—C33—C34	−54.1 (2)
O8—C9—C13—C1	59.12 (16)	C9—C13—C33—C34	70.1 (2)
N1—C9—C13—C1	179.81 (13)	C38—C33—C34—C35	0.5 (3)
C10—C9—C13—C1	−63.34 (16)	C13—C33—C34—C35	−177.78 (18)
O8—C9—N1—C14	13.6 (2)	C33—C34—C35—C36	0.1 (3)
C13—C9—N1—C14	−108.74 (18)	C34—C35—C36—C37	−1.1 (4)
C10—C9—N1—C14	133.19 (16)	C35—C36—C37—C38	1.4 (4)
C9—N1—C14—C15	178.85 (16)	C36—C37—C38—C33	−0.7 (3)
N1—C14—C15—C16	1.3 (3)	C34—C33—C38—C37	−0.2 (3)
N1—C14—C15—C20	179.83 (17)	C13—C33—C38—C37	178.19 (19)

### Hydrogen-bond geometry (Å, °)

**Table:**

*D*—H···*A*	*D*—H	H···*A*	*D*···*A*	*D*—H···*A*
O1—H1O···N1	0.94	1.73	2.608 (2)	154
O2—H2O···O3^i^	0.97	1.67	2.637 (2)	177
O5—H5O···O6^ii^	0.97	1.69	2.651 (2)	174
O6—H6O···O4	0.98	1.65	2.617 (2)	173
N12—H12A···O3	0.93	1.77	2.697 (2)	172
N12—H12B···O5	0.94	1.77	2.709 (2)	173

Symmetry codes: (i) −*x*+1, −*y*+1, −*z*; (ii) −*x*+1, −*y*+1, −*z*+1.
